# Comparative assessment of crestal bone level around non-hydrophilic and hydrophilic implants after immediate extraction: An *in vivo*study

**DOI:** 10.6026/973206300222671

**Published:** 2026-04-30

**Authors:** K Seethal, Deepesh Kumar Gupta, Mahendra Anant, Anumeha Jha, Ambika Thakur

**Affiliations:** 1 Department of Oral and Maxillofacial Prosthodontics and Implantology, Government Dental College, Raipur, Chhattisgarh, India;

**Keywords:** Hydrophilic implant, non-hydrophilic implant, immediate implant placement, crestal bone level, SLA, surface modification, SLActive

## Abstract

Immediate implant placement requires early healing and preservation of crestal bone; however, limited evidence exists regarding the comparative effect of hydrophilic implant surfaces on early bone healing and crestal bone level changes. Therefore, it is of interest to evaluate crestal bone level changes around hydrophilic and non-hydrophilic dental implants placed in immediate extraction sites. Hence, a total of 30 implants were allocated into two groups. Following atraumatic extraction, implants were placed, and crestal bone levels were assessed at baseline, 1, 3, and 6 months. The hydrophilic group demonstrated greater early bone-forming activity with reduced crestal bone changes at 1 month compared to the non-hydrophilic group. However, no statistically significant difference was observed between the groups at 3 and 6 months. Hydrophilic implants may therefore enhance early crestal bone formation in immediate extraction cases.

## Background:

Hydrophilic dental implants have better wettability as compared to non-hydrophilic implants due to increased interaction between the surface of the implant and surrounding structures [1]. The bioactive implant surfaces showed better early implant stability in D3-D4 bone as compared to conventional surfaces, with more consistent ISQ values and a higher proportion of implants suitable for early functional loading, highlighting their advantage in improving the transition from primary to secondary stability in compromised bone conditions [2]. Burser *et al.* have reported that modified SLA implants exhibit greater contact at the bone implant level in the initial 2 and 4 weeks of healing [3]. According to Zhao *et al.* hydrophilic dental implants enhance surface wettability; improve cell response and osseointegration, which contributes to better clinical outcomes [4]. Lang *et al.* also demonstrated that hydrophilic implants have achieved significantly early bone to implant contact than non-hydrophilic surfaces after 14-28 days of healing [5]. Canullo *et al.* in their study found that bioactive hydrophilic surfaces may show a slight improvement in implant stability (ISQ values) around three months after placement, suggesting a potential benefit during the later phase of early osseointegration [6]. de Moraes Ferreira *et al.* in their study on hydrophilic and hydrophobic implant surface found that there was no relevant clinical impact on survival and marginal bone changes after one year of follow-up [7]. Therefore, it is of interest to report that if any crestal bone changes occur due to the hydrophilicity of implants in the immediate extraction cases.

## Methodology:

This single-centre *in vivo* study was conducted at the Government Dental College, Raipur, Chhattisgarh, in eligible patients between May 2022 to March 2024. Follow-up visits were scheduled at 0, 1, 3 and 6 months after fixture placement. After being approved by the ethical committee (IEC Proposal No: 4747/ GDC/Ethical Committee/2022) and the Scientific Committee (S. No: 2952/GDC/Scientific Committee), the study was conducted. Systemically and periodontally healthy individuals of either gender, aged between 18 and 60 years, presenting with a non-restorable tooth with a minimum of 4mm of apical bone in the extraction socket were included in the study. Individuals with systemic conditions such as diabetes mellitus or pregnancy, smokers, periodontal diseases including periapical pathology, narrow alveolar ridge, periodontitis, etc., were excluded from the study. Final case selection was performed according to Kan *et al.* classification [8]. Random allocation of patients was done into two groups. In group 1 patients, non-hydrophilic implants were placed, and in Group 2, Patients received a hydrophilic implant. Atraumatic extraction and implant placement were done as per standard protocol.

## Crestal bone level evaluation:

Crestal bone level was measured using RVG long cone paralleling technique with XCP positioning system. The amount of changes in the crestal bone level was recorded as per schedule using RVG's software. The alveolar crest and implant shoulder were used as reference and the distance between the two reference points was measured at the mesial and distal aspects digitally. For each patient, the mean values were calculated and recorded.

## Statistical analysis:

A non-parametric test was used for the statistical analysis as the data distribution was not normal. To compare the two groups in terms of crestal bone level (CBL) at each point Wilcoxon Mann-Whitney test was applied. The Friedman test was used for the assessment of the changes in the crestal bone level within the group over time, and generalized estimating method was used for evaluating the difference in the CBL changes of both groups.

## Results and Discussion:

In the hydrophilic group, crestal bone level at the mesial and distal aspect showed a slight increase from surgery to 1 month, followed by a reduction at 6 months. In contrast, the non-hydrophilic group demonstrated a continuous decrease in both mesial and distal crestal bone levels from surgery to 6 months (Table 1 - see PDF). The present study was directed towards evaluating the changes in bone level at the crestal part in hydrophilic and non-hydrophilic implant placed in immediate extraction cases, as little data was available in the literature. The hypothesis was rejected as there was an increased bone-forming activity at the end of the first month in the immediately placed hydrophilic implant at the crestal region. As compared to a non-hydrophilic implant, crestal bone level changes in terms of increased bone forming activity were observed at the end of the first month in an immediately placed hydrophilic implant. Vashisht *et al.* evaluated twenty implants and divided them into SLA hydrophobic and SLA hydrophilic groups. Although both groups demonstrated crestal bone loss over time, hydrophilic implants showed significantly less bone loss, indicating better crestal bone preservation and suitability for early loading [9]. It is well documented that due to the surface changes in the hydrophilic surface, there might be selective attraction of proteins which exerts VEGF (Vasculo Endothelial Growth Factor) signalling, and other genes associated with angiogenesis and neurogenesis are upregulated, whereas negative regulators of angiogenesis are downregulated [10]. For osteogenesis, BMP2K (Bone morph genic protein 2 inducible kinase) are both upregulated on hydrophilic surfaces on the 7th day, whereas on the non-hydrophilic surface it happens on the 14th day [10]. The degree of osseointegration was superior at the end of the first month, with bone implant contact of 48.3% on the hydrophilic surface and 32.4% in non-hydrophilic surface [5]. Buser *et al.* and Schwarz *et al.* in their research found that SLActive implants showed increased affinity of blood clot to the surface of the implant, better angiogenesis with improved bone implant contact within one month of bone healing [3, 11]. Horizontal reduction at central sites was comparatively limited, with a median loss of 0.3 mm (3.8%), although variations up to 3.4 mm (49.5%) were observed. Conversely, proximal sites showed a markedly reduced degree of bone resorption in both vertical and horizontal dimensions [12]. The decrease in crestal bone level on the 3rd and 6th months was found to be greater with non-hydrophilic implant as compared to hydrophilic, but the difference was not statistically significant [5, 13]. The comparative evaluation of crestal bone levels in immediate extraction cases showed no statistically significant difference between hydrophilic and non-hydrophilic implants, although hydrophilic implants showed slightly better preservation of crestal bone [14, 15]. Significant progress has been achieved in the development of advanced dental implant surface technologies. These innovations have enhanced clinical outcomes, allowing for reliable rehabilitation with high success and predictable survival rates, even in complex cases [16, 17].

## Conclusion:

In our study, it was found that a hydrophilic implant surface helped in early healing and reduction in crestal bone loss. Enhanced bone deposition activity at the end of 1st month makes the hydrophilic implant more helpful in immediate implant cases and in compromised clinical situations.

## Financial support and sponsorship:

Nil

## References

[1] Rupp F (2018). Dent Mater..

[2] Canullo L (2024). Clin Oral Investig..

[3] Buser D (2004). J Dent Res..

[4] Zhao G (2005). J Biomed Mater Res A..

[5] Lang NP (2011). Clin Oral Implants Res..

[6] Canullo L (2025). Oral Health Prev Dent..

[7] De Moraes Ferreira AC (2025). J Dent..

[8] Kan JYK (2011). Int J Oral Maxillofac Implants..

[9] Vashisht K, Rani S (2023). Int J Prosthodont Restor Dent..

[10] Donos N (2011). Clin Oral Implants Res..

[11] Schwarz F (2009). J Biomed Mater Res B Appl Biomater..

[12] Chappuis V (2013). J Dent Res..

[13] Lai HC (2009). Clin Oral Implants Res..

[14] Yang Y (2026). Oral Health Prev Dent..

[15] Fan L (2025). Medicine (Baltimore)..

[16] Kunrath MF (2022). Nanomaterials (Basel)..

[17] da Silva RA (2020). Biomed Res Int..

